# Editorial: Hypertension in the Elderly

**DOI:** 10.3389/fcvm.2021.645580

**Published:** 2021-02-24

**Authors:** Antonio Coca, Michel Burnier

**Affiliations:** ^1^Hypertension and Vascular Risk Unit, Department of Internal Medicine, Hospital Clínic, University of Barcelona, Barcelona, Spain; ^2^Service of Nephrology and Hypertension, Lausanne University Hospital and University of Lausanne, Lausanne, Switzerland; ^3^Hypertension Research Foundation, St-Légier, Switzerland

**Keywords:** early vascular aging, arterial stiffness, cognitive decline, vascular dementia, antihypertensive drugs, treatments adherence, persistence on treatment

Although the cardiovascular (CV) risk increases with age leading to negative health consequences such as stroke, coronary artery disease, and dementia, the aging process takes a more rapid course in some individuals. These subjects suffer the so called Early Vascular Aging (EVA) syndrome, which core is arterial stiffness due to the structural changes of the large elastic arteries induced by aging but accelerated by CV risk factors such as hypertension, dyslipidemia, obesity, and type 2 diabetes, sharing a common pathophysiological pattern with chronic inflammation and oxidative stress. These changes are associated with neuronal dysfunction leading to cognitive decline and dementia, among many other health problems. No doubt that many factors associated with aging may contribute to reduce the adherence and persistence of long-term therapies prescribed to control CV risk factors.

In this collection “hypertension in the elderly,” **Nilsson** reviews several aspects of the EVA syndrome including its definition, influencing factors and determinants, pathophysiology, organ damage, and treatment. Hypertension is one of the main factor modifying arterial hemodynamics, but non-hemodynamic factors such as abnormalities of glucose and lipid metabolism, oxidative stress, and inflammation are important protagonists of the EVA syndrome which pathophysiological hallmark is arterial stiffness easily detected by an increased pulse wave velocity in large arteries.

The article by Laurent et al. goes in deep on the stiffening process in large arteries in elderly subjects (aged >65 years), and remind readers that the main reason for measuring arterial stiffness in clinical practice in elderly hypertensive patients is based on the demonstration that arterial stiffness and wave reflections are significant predictors of cardiovascular events. Reference values for the European population have been published and evidence-based cut-off values for different age strata are now available for use in daily clinical practice. The complete review by the authors include an approach to the cellular and molecular mechanisms of arterial stiffening during the aging process, the consequences of this structural change on central systolic and pulse pressures, and the effects of arterial pulsatility on target organs. One on the most interesting aspects addressed by the review is the methodology for measuring arterial stiffness, central pulse pressure and wave reflection. Finally, the chance of destiffening large arteries by new pharmacological treatments is widely discussed, with a specific mention of this aspect in the very elderly population.

**Sierra** reviews the consequences of the EVA syndrome, stiffening, and changes in the small arteries of the brain induced by the association of aging and hypertension. Today, it is well-established that hypertension, commonly associated to obesity, and glucose and lipid abnormalities, induces functional and structural changes in the small arteries penetrating into the brain leading to silent subcortical and periventricular white matter lesions, microbleeds, and lacunar infarcts, the pathological substrates of the early cognitive decline that may evolve to dementia. Not only vascular dementia is associated with hypertension and CV risk factors but also progression of Alzheimer disease is negatively affected by high blood pressure values. The most important issue related to this problem is that treatment and control of hypertension with antihypertensive drugs may protect the brain and delay the progression of cognitive decline to established dementia. As mentioned by Sierra C, although in middle-aged hypertensive patients a systolic blood pressure <130 mmHg target for cognitive decline prevention seems effective in at least two clinical trials, we do not know the optimal blood pressure target in the elderly population.

The article by Burnier et al. faces the very relevant clinical problem of the adherence to treatment. In contrast to the general belief, patients older than 65 years often have a better adherence to medications than younger patients do. Yet, in very old patients (>80 years), the prevalence of non-adherence increases due essentially to the cognitive decline, depression and health believes. In the elderly, one interesting new aspect to consider is the process of deprescribing, withdrawing unnecessary drugs interfering with the adherence to important medications.

In summary, the articles of this Research Topic discuss some relevant issues in the field of hypertension in the elderly. The main messages of these articles are: (1) arterial stiffening due to aging may start much earlier than believed and hence, would need an early detection, (2) despite the absence of large randomized controlled trials demonstrating the benefits of treating hypertension on the prevention of cognitive decline, there is now increasing evidence that lowering any elevated blood pressure provides more neurological benefits than harms even in very elderly patients, and (3) drug adherence is an important issue for all patients treated for hypertension. Yet, drug adherence is often better in elderly patients than in younger patients, except perhaps for very elderly.

The elderly population is the largest to be treated for hypertension. Several additional issues could have been discussed. Thus, for example, the recommendations on the levels of blood pressure above which elderly, and particularly very old patients, should be treated and the target blood pressures to be reached on treatment are still variable among international guidelines. Another point is the definition and assessment of frailty, a concept that is now widely recommended to evaluate the pertinence of starting any antihypertensive therapy in very old patients. Today, there is a lack of standardization and knowledge among physicians on the criteria to be used to assess frailty. At last, the impact of antihypertensive drugs on the quality of life of treated elderly patients could be reappraised in the light of new clinical data.

## Author Contributions

AC and MB prepared the editorial. All authors contributed to the article and approved the submitted version.

## Conflict of Interest

The authors declare that the research was conducted in the absence of any commercial or financial relationships that could be construed as a potential conflict of interest.

